# Delocalized Surface State in Epitaxial Si(111) Film with Spontaneous √3 × √3 Superstructure

**DOI:** 10.1038/srep13590

**Published:** 2015-08-28

**Authors:** Jian Chen, Yi Du, Zhi Li, Wenbin Li, Baojie Feng, Jinlan Qiu, Peng Cheng, Shi Xue Dou, Lan Chen, Kehui Wu

**Affiliations:** 1 Beijing National Laboratory for Condensed Matter Physics and Institute of Physics, Chinese Academy of Sciences, Beijing 100190, China; 2 Institute for Superconducting and Electronic Materials (ISEM), University of Wollongong, Wollongong, NSW 2525, Australia; 3 Collaborative Innovation Center of Quantum Matter, Beijing 100871, China

## Abstract

The “multilayer silicene” films were grown on Ag(111), with increasing thickness above 30 monolayers (ML). Scanning tunneling microscopy (STM) observations suggest that the “multilayer silicene” is indeed a bulk-like Si(111) film with a (√3 × √3)R30° honeycomb superstructure on surface. The possibility for formation of Si(111)(√3 × √3)R30°-Ag reconstruction on the surface can be distinctively ruled out by peeling off the surface layer with the STM tip. On this surface, delocalized surface state as well as linear energy-momentum dispersion was observed from quasiparticle interference patterns. Our results indicate that a bulklike silicon film with diamondlike structure can also host delocalized surface state, which is even more attractive for potential applications, such as new generation of nanodevices based on Si.

Silicon is the basis of modern microelectronics industry. Unlike carbon that exhibits both sp^2^ and sp^3^ allotropes represented by graphite and diamond, silicon has only diamond-like structure with sp^3^ bonding in nature. Recently, silicene, a single sheet of Si atoms arranged in honeycomb lattice with hybridized sp^2^/sp^3^ bonding, has been predicted^1^,^2^ and successfully fabricated^3^, ^4^, ^5^, ^6^, ^7^, ^8^, ^9^, ^10^, ^11^, ^12^, ^13^. Similar to graphene, the band structure of silicene hosts Dirac fermions and hence exotic properties for potential applications such as quantum spin Hall effect and spintronics devices^2^. However, recent studies revealed strong influence of the substrate on the electronic structure of monolayer silicene, and that the Dirac state could no longer exist in monolayer silicene on metal substrate^4^,^14^, ^15^, ^16^, ^17^, ^18^. This is a serious challenge to any further research and application of silicene.

In this Letter, we overcome this problem by pointing out that delocalized electronic surface state can exist on a bulk-like Si(111) film surface. The study was motivated by the previous report of “multilayer silicene” film on Ag(111)^8^,^19^,^20^. We performed a comprehensive study on the “multilayer silicene” films grown on Ag(111), with increasing thickness above 30 monolayers (ML) by scanning tunneling microscopy (STM) and spectroscopy (STS). Our original intention was to find the “critical thickness” for the “multilayer silicene” film to transfer from graphite-like structure to diamond-like structure, as it eventually should do. We found, however, that the “multilayer silicene” is most likely a bulk Si(111) film with diamond structure from the beginning. Strikingly, in this specific case the film always exhibits a (√3 × √3)R30° honeycomb superstructure on the surface, in contrast to the well-known 7 × 7 reconstruction on bulk Si(111) surface. The possibility for the (√3 × √3)R30° to be a Ag-induced Si(111)(√3 × √3)R30°-Ag reconstruction, as suggested recently by Shirai *et al.*^21^, had been distinctively ruled out by experimentally peeling off the surface layer at liquid nitrogen temperature using the STM tip. More interestingly, quasiparticle interferences (QPI) patterns were observed for all films with different thicknesses, and linear energy-momentum dispersion has been deduced. Such (√3 × √3)R30° reconstruction, and delocalized surface state have never been observed on surface of bulk silicon in the intensive study in the last several decades. The bulk-like Si film with delocalized surface state is fascinating for potential nano device applications as compared with monolayer silicene, since it is easier to obtain, substrate effects are avoided, and directly compatible with the silicon microelectronic industry.

## Results and Discussions

**Figure 1:**
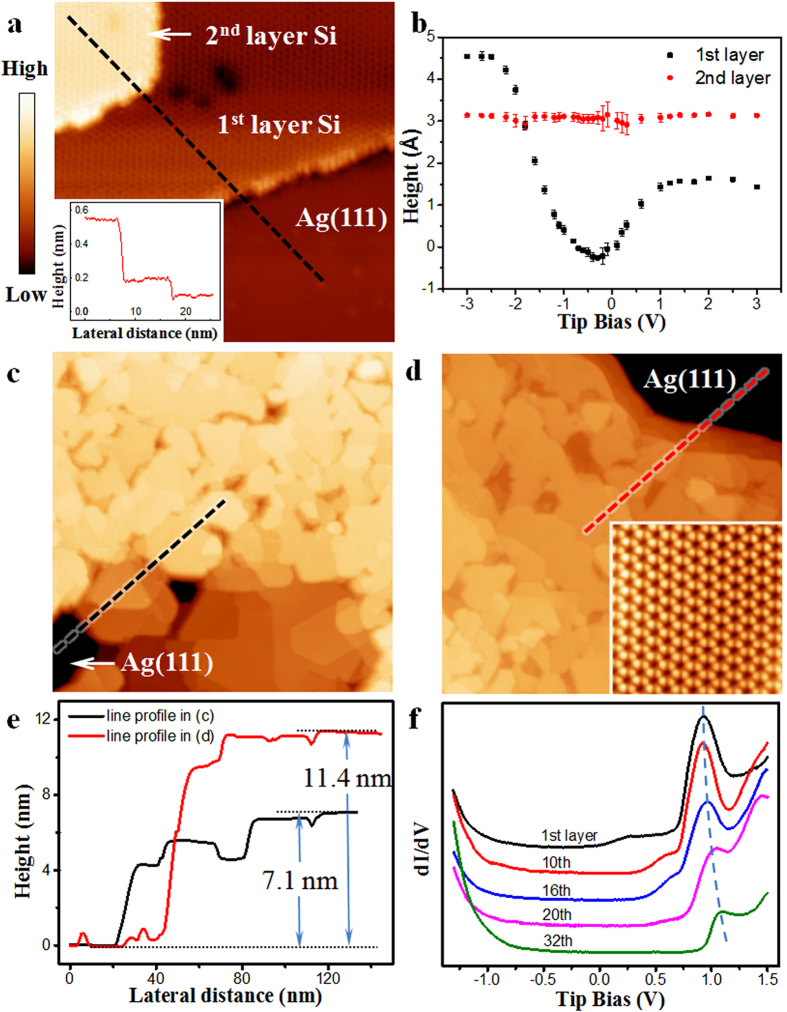
The STM observation of Si film with different layers on Ag(111) surface. (**a**) STM image (V_tip_ = 0.9 V, I = 100pA, 20 × 20 nm^2^) of the first two √3 layers on Ag(111). (**b**) The heights of the first two √3 layers as a function of tip bias. (**c**,**d**) STM images (V_tip_ = −1.0 V, I = 100pA, 200 × 200 nm^2^) of multilayer Si films with different thickness. The inset is high resolution STM image (V_tip_ = −0.5 V, I = 100 pA, 6 × 6 nm^2^) on the top surface of the film in (**d**), being √3 × √3 reconstructed. (**e**) The line profiles across the substrate to the Si films along the dash lines in (**c**) and (**d**), respectively. The measured film thicknesses are indicated. (**f**) dI/dV curves obtained on surface of Si films with different thickness. The curves are vertically shifted for clarity.

A monolayer silicene film grown on Ag(111) surface exhibits a variety of different structural phases such as 4 × 4^3^,^22^,^23^,^24^,^25^, √13 × √13^3^,^4^, √7 × √7^3^,^25^, 2√3 × 2√3^24^,^25^ (with respect to Ag(111) surface lattice) and √3 × √3^3^,^6^ (with respect to silicene 1 × 1). On the other hand, “multilayer silicene” films only exhibit √3 × √3 honeycomb superstructure, as shown in Fig. 1(a). The line profile in Fig. 1(b) shows that the apparent height of the first √3 layer varies significantly from 0 to 0.48 nm with bias, whereas the height of second √3 layer, 0.31 nm, is almost constant. This can be explained by the fact that the local density of states (LDOS) is different on the √3 layer surface and the Ag(111). On the other hand, the almost constant height for thicker √3 layers indicates the same LDOS for different √3 layers. We have analyzed the layer distance for different layer thickness, and we obtained strictly 0.31 ± 0.02 nm, which coincides with the layer distance in bulk Si(111) layers.

**Figure 2:**
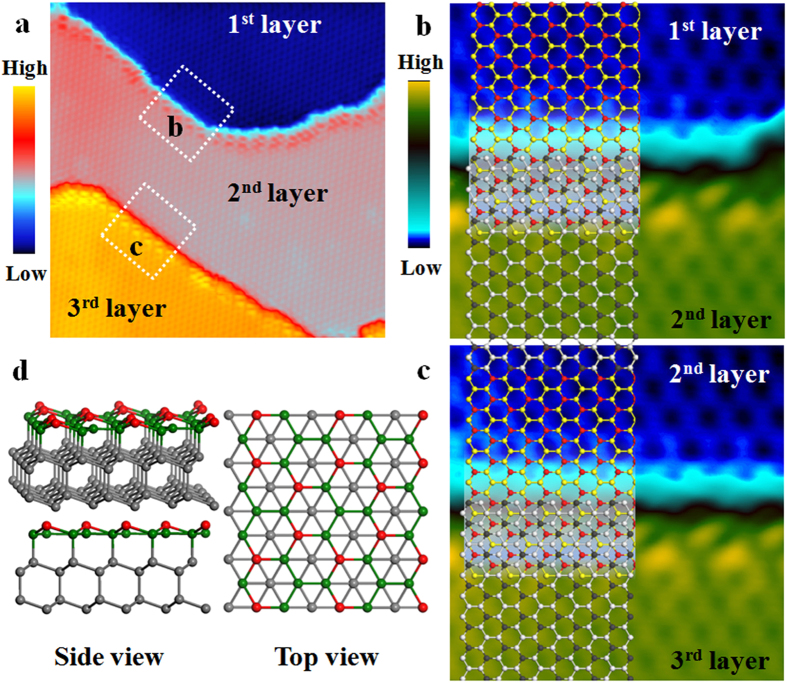
Stacking sequence of Si film on Ag(111). (**a**) The STM image (V_tip_ = 1.2 V, I = 100 pA, 25 × 25 nm^2^) of Si film with three continuous terraces. (**b**) and (**c**) The high resolution STM images of the areas labeled by white squares in (**a**). The atomic model of the √3 × √3 of silicene is superimposed, and the stacking sequence of the neighboring Si layers is ABC stacking. (**d**) The side and top view of the atomic model of Si film with (√3 × √3)R30° reconstruction. The red and green balls represent silicon atoms with different buckling heights in first layer of Si film, respectively. The gray balls represent silicon atoms below first layer.

The “multilayer silicene” films with thickness more than 30 layers (see Fig. 1(c,d), the thickness can be determined by the line profiles) were prepared. We observed, strikingly, that identical √3 × √3 honeycomb superstructure persists on the surface up to the maximum thickness that we have obtained (inset in Fig. 1(d)). On the other hand, when we superimpose the atomic models of √3 × √3 phase of silicene on the atomically resolved images near the step edges (Fig. 2(b,c)), the ABC stacking sequence of Si layers is always found, for all different layer thickness.

The strict ABC stacking, and layer distance of 0.31 nm, are beyond our original expectation for a “multilayered silicene”. If the neighboring silicene layers interact with weak van der Waals force just like graphite, the layer distance should be notably larger than 0.31 nm, which is the distance between Si(111) planes with strong covalent bonds. And one should be able to observe other stacking sequence, or twisting between neighboring layers due to the weak interaction between layers. Both the above two facts point to a conclusion that the so-called “multilayer silicene” is actually a bulk-like Si(111) film, but with √3 × √3 honeycomb reconstruction on its surface, the atomic model being shown in Fig. 2(d). The above model is also supported by our Raman measurements. It was reported that the Raman features of monolayer silicene are different from bulk Si^26^. But our Raman spectroscopy on “multilayer silicene” film only shows a 520 cm^−1^ peak identical to bulk Si (see Fig. S1 in the Supplementary Information). If the film structure is graphite-like, one should expected even stronger deviation of the spectrum from bulk Si due to the overlapping of signals from different silicene layers. So far, we can not figure out any other possible silicon structure that could own the above three properties. Therefore, in the following we will refer to our “multilayer silicene” film as a bulk-like Si(111) film.

**Figure 3:**
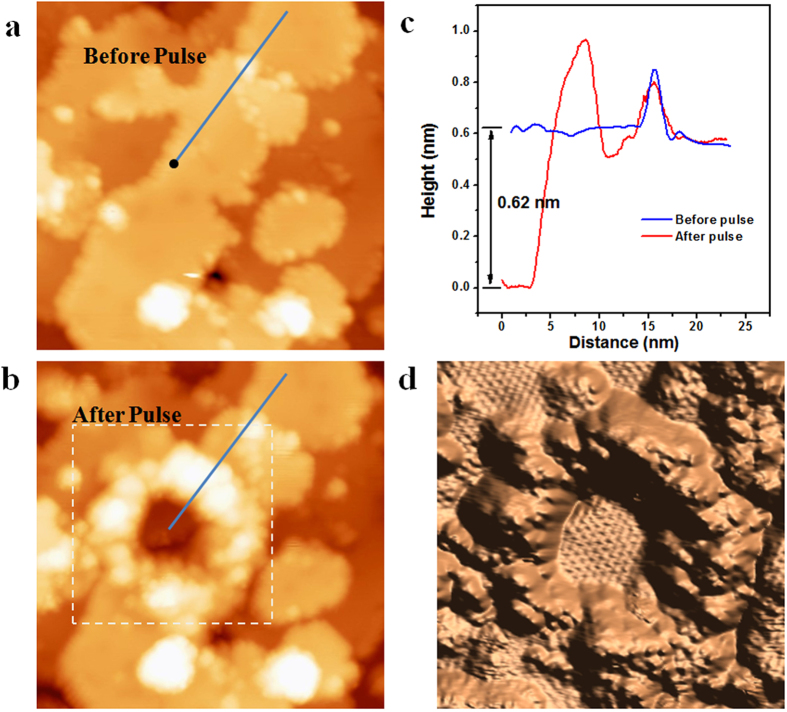
Peeling off the surface layers of Si film by STM tip. (**a**,**b**) The STM images of same area (V_tip_ = 1.0 V, I = 100 pA, 40 × 40 nm^2^) taken before and after applying a series of bias pulses (−5 V, 50 ms) at the position marked by the black dot in (**a**), respectively. (**c**) The line profiles along the blue lines in (**a**,**b**) respectively. (**d**) 3D version of the dotted box in (**b**) which indicates the atomic structures of top layer.

Recently, Shirai *et al.*^21^, suggested that the √3 × √3 structure observed in our film could be actually a Si(111)(√3 × √3)-Ag surface, formed due to the segregation of Ag to the Si surface. Indeed, in STM experiments, our √3 × √3 phase and the Si(111)(√3 × √3)-Ag surface^27^ appear very similar. Fortunately, we can distinctively rule out this possibility, as follow. We apply a series of repeated bias pulses to the tip on the surface of √3 × √3 phase, and the top layer beneath the tip is damaged and removed, leaving a pit inside which the underneath layer is exposed, as shown in Fig. 3(a,b). The line profiles shown in Fig. 3(c) indicate that the area inside the pit is about 0.62 nm lower than the original surface, corresponds removal of 2 layers from the surface. The 3D, derivative STM image in Fig. 3(d) shows the atomic structures of the exposed layer, which is identical to the top layer and exhibits a √3 × √3 reconstruction. As we know, the Si(111)(√3 × √3)-Ag surface consists of only one layer of Ag atoms on the top surface of pure Si(111) substrate, and it forms at temperature above 700K^27^, ^28^, ^29^. Supposing that our √3 × √3 structure comes from Ag-Si(111)-(√3 × √3)R30°, the field evaporation induced by the bias pulse^30^,^31^ remove the top layer at liquid nitrogen temperature, and we should not observe the √3 × √3 reconstruction on the underneath layer. Therefore, our √3 × √3 reconstruction is not the Ag-Si(111)-(√3 × √3)R30°, but is an intrinsic structure of pure Si.

Additionally, from STM images (Fig. 1(c,d)), we found the growth mode of multilayer silicene on Ag(111) is Volmer-Weber growth, which lead to the formation of islands. In other word, no matter how many layers growth of Si on Ag(111), there is still area of Ag(111) uncovered by Si. So the *ex-situ* XPS measurements always indicate the Ag signals (Fig. S2 in supplementary information), and do not provide the strong evidence to exclude the possibility of Ag on Si surface. But there are still other differences between our √3 × √3 and Si(111)(√3 × √3)-Ag. For example, although both surfaces exhibit a structural phase transition at low temperature, the transition temperature is drastically different: 30–40 K for our √3 × √3 phase^32^, and above 150 K for Si(111)(√3 × √3)-Ag^28^. Another example is that J. Zhuang *et al.* reported the *in-situ* Raman spectroscopy on silicene with √3 × √3 phase^33^, which indicate the 2D mode of silicene and is different with Si(111)(√3 × √3)-Ag. Similar results were also reported by P. De Padova *et al.*^34^.

Based on the above picture, a key question would be why a √3 × √3 reconstruction is formed on the surface, instead of the well-known 7 × 7, 5 × 5, or 2 × 1 on Si(111)^35^,^36^. This can be qualitatively understood as follow. On the surface of bulk-terminated Si(111)1 × 1, there is one dangling bond per unit cell due to the symmetry breaking along Z direction. Forming a 7 × 7 reconstruction can lower the number of dangling bonds and stabilize the surface^37^, but it requires a temperature higher than 800 °C to overcome the high energy barrier for its formation. However, in our experiments the sample temperature is lower than 300 °C. If the substrate temperature is higher than 350 °C, the Si film would desorb completely^3^. In this case the system has to choose other solution, such as the 2 × 1 reconstruction found in cleaved Si(111) surface. It is still unclear why we should see the (√3 × √3)R30° reconstruction instead of the better known 2 × 1. However, both (√3 × √3)R30° and 2 × 1 involves changes in the bond angle/distance among surface atoms. The stability of (√3 × √3)R30° and 2 × 1 may be quite close, and it could result in the choice of (√3 × √3)R30° in this specific case. The stability of Si(111) film with √3 × √3 reconstruction on Ag(111) has been confirmed by density function theory (DFT) calculations recently^38^.

Typical dI/dV curves obtained on surfaces with different thickness, as shown in Fig. 1(f), reveal similar features: a pronounced peak at positive bias 0.9V–1.1 V and a DOS onset at negative bias 0.7–0.9 V. While the film thickness increases, the positions of the peak and LDOS onset both shift slightly to the right. The differential conductance (dI/dV) maps show obviously standing waves corresponding to quasiparticle interference (QPI) patterns. Such QPI patterns can be observed on Si films of all different thicknesses, even above 30 ML. This indicates that the metallic surface state on our Si(111) surface is delocalized, and it should originate from the √3 × √3 superstructure on the surface, and not from the Ag(111) substrate.

**Figure 4:**
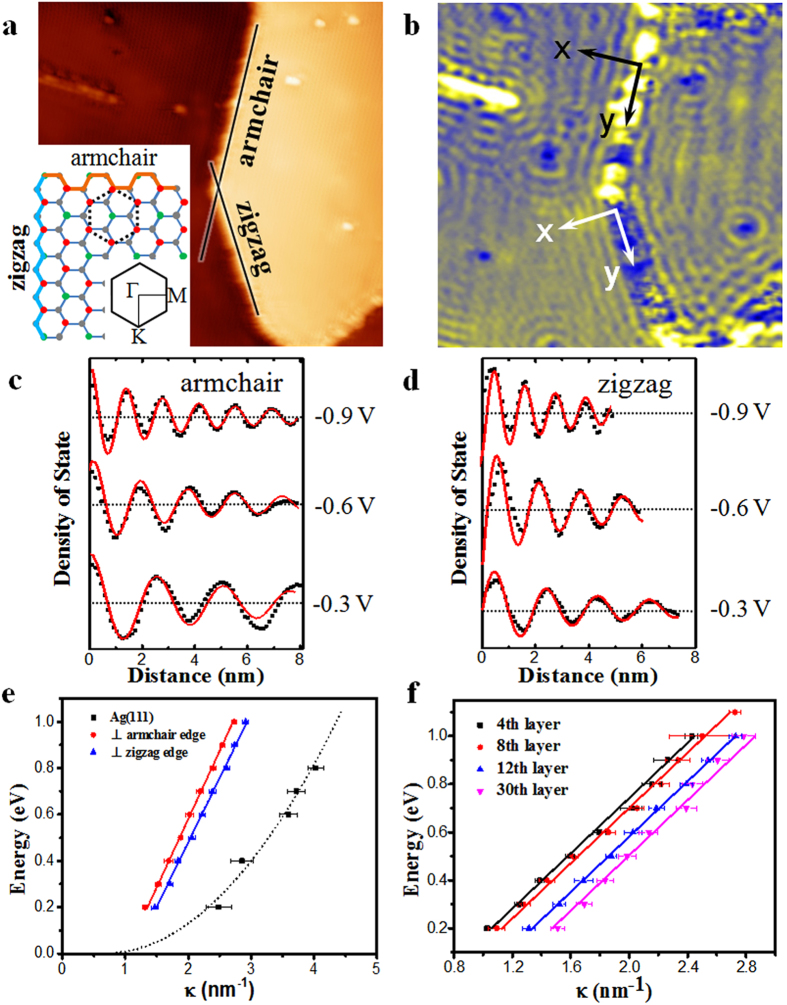
Quasiparticle interference patterns on Si film with different layers. (**a**) STM image (V_tip_ = −0.4 V, I = 100 pA, 45 × 45 nm^2^) obtained on top of Si film of 20 ML, containing an island with both armchair and zigzag step edges. Inset: the atomic model of √3 × √3 superstructure of Si. The black dash hexagon shows a honeycomb unit cell. The first Brillouin zone of the Si(111) 1 × 1 lattice is also shown. (**b**) dI/dV map (V_tip_ = −0.4 V, I = 200 pA, 45 × 45 nm^2^) of the same area as (**a**) showing obvious standing wave. (**c**,**d**) Line profiles of LDOS along the *x* axis for armchair and zigzag edges labeled in (**b**) at various energies, respectively. The back dash lines are experimental values, and the red lines are the lines fitting to the data. (**e**) Energy-momentum dispersions (E–k) determined from wave length of standing waves from armchair and zigzag edges in (**c**) and (**d**), respectively. The Energy-momentum dispersion obtained from Ag(111) surface state is also shown for comparison. (**f**) E–k curves (armchair edge) obtained on surface of Si films with different thickness on Ag(111) surface.

To quantitatively investigate the energy-momentum dispersion of the surface state, we focus on the standing waves around step edges. There are two types of step edges: zigzag and armchair, as exemplified in Fig. 4(a). The dI/dV map (shown in Fig. 4(b)) taken at same area as Fig. 4(a) shows QPI patterns near both step edges. We plot the dI/dV intensity as a function of the distance from the step edges at various energies, and examples are shown in Fig. 4(c,d). Here the direction normal to the step edge is defined as *x* and the direction parallel with the step edge as *y*. The dI/dV signal has been averaged in *y* direction to maximize the signal-to-noise ratio. Clear oscillatory and decaying behavior of dI/dV signal along *x* axis is observed. The wavelength varies with the bias voltage, in other word, energy. We drew E(κ) curves to deduce the energy-momentum dispersion relation with 2κ=|q| (q is scattering vector). Fig. 4(e) displays examples of the E(κ) curves deduced from standing waves near armchair and zigzag step edges of a 20 ML film, corresponding to dispersions at Γ-K and Γ-M directions of Brillouin zone (BZ), respectively. Fig. 4(f) shows the curves along Γ-K for films with thickness of 4, 8, 12 and 31 ML. All the curves exhibit linear energy-momentum dispersion with the same slopes, but are right shifted with increasing thickness, which coincides well with the right shift of peak position in dI/dV curves shown in Fig. 1(f). A possibility to account for the thickness-dependent energy shift is based on double barrier tunneling junction model^39^. The STM tunneling junction involves two parts: one junction is between the STM tip and the Si film surface, and the other between Si film and Ag(111) substrate. The bias voltage applied between sample and tip will be divided onto two junctions. The magnitude of voltage drop in each junction is proportional to the resistance of junction. When the thickness of Si film increases, the resistance of the junction between Si and Ag also increases. So the voltage drop between the STM tip and the Si film surface decreases. As a result, the measured electronic band will be lowered. We note that a quantitative explanation here is still not available and it needs more theoretical efforts.

The well known surface reconstructions of Si(111), such as 7 × 7 and 5 × 5, are metallic due to a half-filled dangling bond in surface Si adatoms . However, the metallic surface states are localized because the distance between the dangling bonds is too long for their electron orbits to overlap. In the case of √3 × √3 reconstruction, the bonds in the surface consist of mixed sp^2^ and sp^3^ orbits, which are more extended is space, while the distance between them are short enough (one lattice constant). Therefore, the bonds could overlap to form the delocalized surface states. Indeed, DFT calculations also reproduced the linear-dispersed surface states on √3 × √3 reconstruction of Si(111) film on Ag(111)^38^.

It has long been appreciated theoretically that the honeycomb lattice and mapping of the sub-lattice degree of freedom to a pseudospin is represented by the Dirac equation for electrons bound to the lattice. Note that the existence of Dirac fermions in artificially constructed molecular graphene by CO molecules on Cu(111) surface has also been confirmed^40^. The honeycomb arrangement of dangling bonds (upper buckled Si atoms) in √3 × √3 superstructure, which is similar with graphene, may also induce the Dirac state. If it is true, the linear dispersion of the new surface states might originate from the elastic scattering of quasiparticles within the same Dirac cone (usually called intravalley scattering^41^). The slopes of curves give the Fermi velocity V = (0.90 ± 0.05) × 10^6^ m/s (Γ-K direction) and (0.83 ± 0.05)  ×  10^6^ m/s (Γ-M direction), respectively. The energy position of Dirac point (DP), determined by the κ = 0 energy intercept, is from −0.4 eV to −0.7 eV corresponding to films with different thickness. Additionally, Dirac-like electronic state has been reported by P. De Padova *et al.* and J. Zhuang *et al.* in the single or a few layer silicene with √3 × √3 structure on Ag(111) by angle-resolved photoelectron spectroscopy (ARPES)^33^,^42^.

## Conclusion

Our present work reveals that the “multilayer silicene” on Ag(111) is most likely a bulk Si(111) film with (√3 × √3)R30° honeycomb superstructure on its surface. We observed delocalized surface states on this particular system, and deduced linear energy-momentum dispersion. We believe that the unique (√3 × √3)R30° honeycomb structure on the surface is the key for the formation of delocalized surface state. Undoubtedly, if a bulk-like silicon film can also host the similar surface state as monolayer silicene, it will combine the revolutionary concept such as quantum computing with the current microelectronics industry based on Si in a much more attractive an practical may.

## Methods

The experiments were performed in an ultra-high-vacuum chamber (base pressure better than 1.0 × 10^−10^ Torr) equipped with a home-made low-temperature STM. A single-crystal Ag(111) substrate was cleaned by standard sputtering-annealing procedure. Silicon was evaporated from a heated Si wafer with a deposition flux of about 0.3 ML per minute. For the formation of (√3 × √3) phase, the temperature of substrate should be hold at 450 ~ 550 K during Si evaporation. When substrate temperature is about 450 ~ 500 K, the Si coverage should be larger than 1 ML. But when substrate temperature is higher than 500 K, any amount of Si coverage will be OK. The STM observations were performed at liquid nitrogen temperature (77 K) with chemically etched tungsten tip. All the STM data were recorded in the constant-current mode with the bias voltage *V* applied to the tip. The differential conductance (dI/d*V*) maps were extracted from the lock-in signal by applying a modulation of 20 mV at 777 Hz to the tip bias.

## Additional Information

**How to cite this article**: Chen, J. *et al.* Delocalized Surface State in Epitaxial Si(111) Film with Spontaneous √3×√3 Superstructure. *Sci. Rep.* **5**, 13590; doi: 10.1038/srep13590 (2015).

## Supplementary Material

Supplementary Information
