# Supplementary material for: Delocalized Surface State in Epitaxial Si(111) Film with Spontaneous √3 × √3 Superstructure
Source: Sci Rep. 2015 Aug 28;5:13590. doi: 10.1038/srep13590 (PMC4551997; doi:10.1038/srep13590)
Supplement: Supplementary Information [file srep13590-s1.doc]

**Delocalized Surface State in Epitaxial Si(111) Film with Spontaneous √3×√3 Superstructure**

Jian Chen^1^, Yi Du^2^, Zhi Li^2^, Wenbin Li^1^, Baojie Feng^1^, Jinlan Qiu^1^, Peng Cheng^1^, Shi Xue Dou^1^, Lan Chen^1,*^ and Kehui Wu^1,3,*^

^1^Beijing National Laboratory for Condensed Matter Physics and Institute of Physics, Chinese Academy of Sciences, Beijing 100190, China. ^2^Institute for Superconducting and Electronic Materials (ISEM), University of Wollongong, Wollongong, NSW 2525, Australia. ^3^Collaborative Innovation Center of Quantum Matter, Beijing 100871, China. *Correspondence and requests for materials should be addressed to: [lchen@iphy.ac.cn](mailto:lchen@iphy.ac.cn) (L. C.) & [khwu@iphy.ac.cn](mailto:khwu@iphy.ac.cn) (K. W)

**Supplementary Information**

1. **Raman spectroscopy**


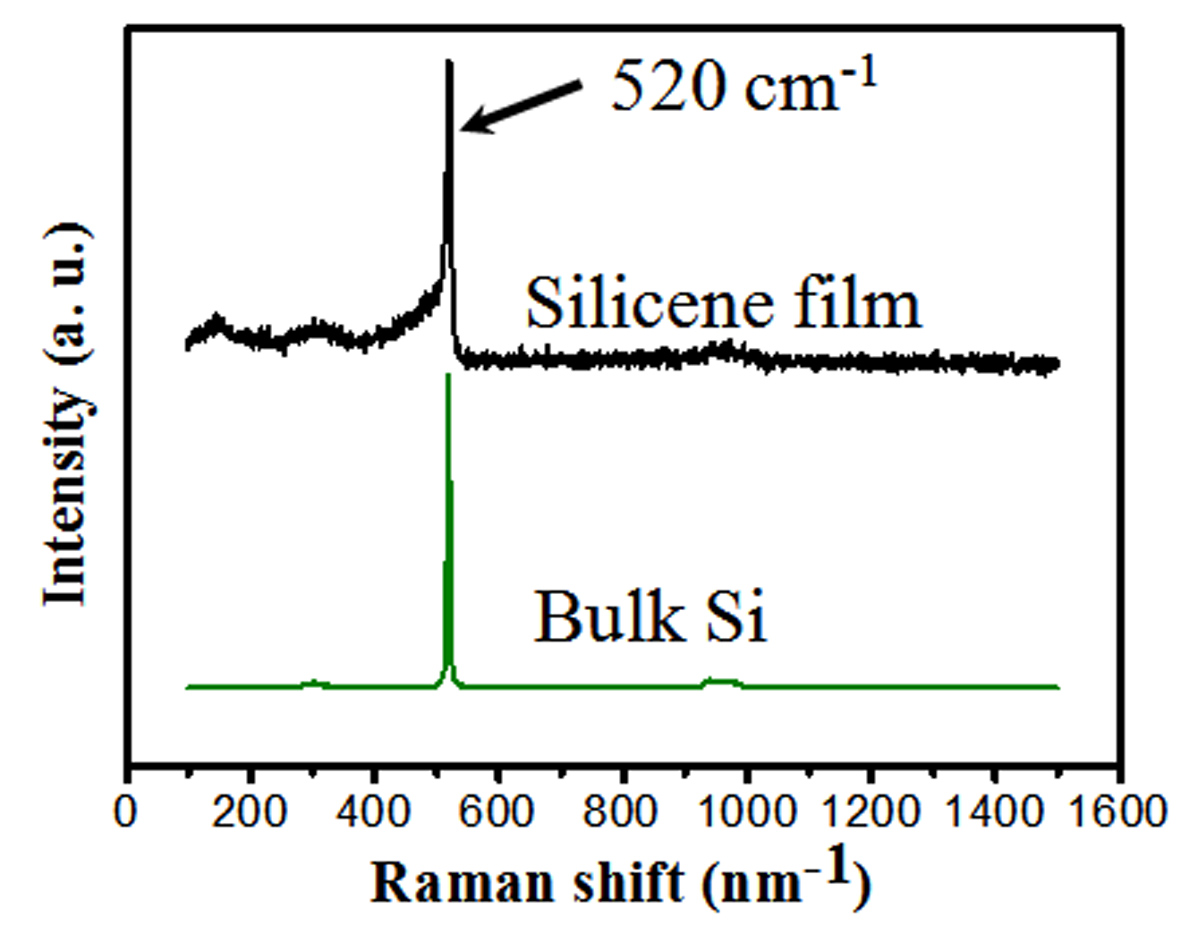


Fig. S1. Micro Raman spectra of Si film with (√3×√3)R30° reconstruction on Ag(111) surface obtained at ∼1.5 kW cm^-2^ and three successive expositions of 40 s. In this experiment, we prepared the Si film with thickness about 10 layers on Ag(111) in UHV chamber first. Then the sample was taken out immediately to perform Raman spectroscopy in air. According to Ref.1, the Si film with (√3×√3)R30° reconstruction on Ag(111) is stable against oxidation in 24 hours without capping. So the ex-situ Raman measurement should be reliable. Spectrum of Bulk Si is included at the bottom as reference.

1. **XPS measurements**

We have done ex-situ X-ray photoelectron spectroscopy (XPS) measurements on multilayer silicene with different thickness. Our XPS results always show the Ag signal, as shown in Fig. S2. That is because the growth mode of multilayer silicene on Ag(111) is Volmer-Weber growth, which lead to the formation of islands. In other word, no matter how many layers growth of Si on Ag(111), there is still area of Ag(111) uncovered by Si. Furthermore, we did observed the decrease of the Ag signal with film thickness, indicating that the Ag signal is not only from the Si surface. As we know, according to IET model, only one layer of Ag atoms is on top of pure Si(111) substrate, so the Ag/Si ratio of Ag-Si(111)-(√3×√3)R30° surface in XPS spectroscopy should be a constant. However, in our XPS results, the Ag/Si ratio decreases very fast while the thickness of Si films increase from 10 nm to 30 nm. Therefore, we believe the Ag signal should come from the Ag(111) substrate.


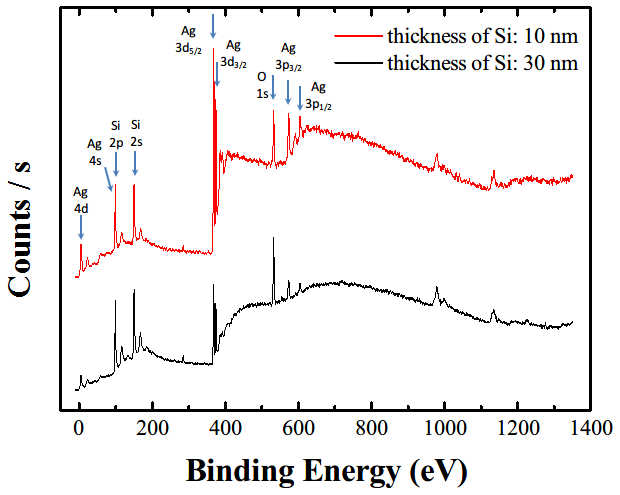


Fig. S2 XPS spectra of **√**3-Si film on Ag(111) surface with different thickness. The curves are vertically shifted for clarity. In this experiment, we prepared the Si film with thickness about 10 nm and 30 nm on one Ag(111) substrate in UHV chamber successively. Then the sample was taken out immediately to perform XPS spectroscopy. Pressure was in the 10^-10^ torr range during the XPS experiments. XPS spectra were recorded by a XPS KRATOS, AXIS Ultra DLD spectrometer with the monochromatized Al Kα X-ray source (hυ = 1486.6 eV) and an X-ray beam of around 1 mm. The analyzer was operated in constant pass energy of 40 eV using an analysis area of approximately 700 μm × 300 μm. The C 1s (285.0 eV) binding energy (BE) was used as internal reference. The spectrometer BE scale was initially calibrated against the Ag 3d_5/2_ (368.2 eV) level.

[1] P. De Padova, C. Ottaviani, C. Quaresima et al., 2D Materials 1, 021003 (2014).
